# Single nucleotide polymorphism (SNP) discovery in duplicated genomes: intron-primed exon-crossing (IPEC) as a strategy for avoiding amplification of duplicated loci in Atlantic salmon (*Salmo salar*) and other salmonid fishes

**DOI:** 10.1186/1471-2164-7-192

**Published:** 2006-07-27

**Authors:** Heikki J Ryynänen, Craig R Primmer

**Affiliations:** 1 Department of Biological and Environmental Sciences, University of Helsinki, P.O. Box 65, FIN-00014 University of Helsinki, Finland; 2 Department of Biology, University of Turku, FIN-20014, Finland

## Abstract

**Background:**

Single nucleotide polymorphisms (SNPs) represent the most abundant type of DNA variation in the vertebrate genome, and their applications as genetic markers in numerous studies of molecular ecology and conservation of natural populations are emerging. Recent large-scale sequencing projects in several fish species have provided a vast amount of data in public databases, which can be utilized in novel SNP discovery in salmonids. However, the suggested duplicated nature of the salmonid genome may hamper SNP characterization if the primers designed in conserved gene regions amplify multiple loci.

**Results:**

Here we introduce a new intron-primed exon-crossing (IPEC) method in an attempt to overcome this duplication problem, and also evaluate different priming methods for SNP discovery in Atlantic salmon (*Salmo salar*) and other salmonids. A total of 69 loci with differing priming strategies were screened in *S. salar*, and 27 of these produced ~13 kb of high-quality sequence data consisting of 19 SNPs or indels (one per 680 bp). The SNP frequency and the overall nucleotide diversity (3.99 × 10^-4^) in *S. salar *was lower than reported in a majority of other organisms, which may suggest a relative young population history for Atlantic salmon. A subset of primers used in cross-species analyses revealed considerable variation in the SNP frequencies and nucleotide diversities in other salmonids.

**Conclusion:**

Sequencing success was significantly higher with the new IPEC primers; thus the total number of loci to screen in order to identify one potential polymorphic site was six times less with this new strategy. Given that duplication may hamper SNP discovery in some species, the IPEC method reported here is an alternative way of identifying novel polymorphisms in such cases.

## Background

The diversification of the variety of molecular markers available has been an important development in the field of genetics over the past two decades [1], with one of the more recent additions to the 'molecular toolbox' being single nucleotide polymorphisms (SNPs): a variant of traditional DNA sequencing which potentially enables high-throughput analysis of numerous independent (mostly) bi-allelic DNA sequence polymorphisms. The increase in the range of molecular markers partly stems from the realisation that no particular marker type is ideal for all situations, and SNPs are no exception to this. Their beneficial features include having a relatively simple mutation model [2,3] and a high abundance in the genome (see e.g. [4]). Furthermore, the fact that SNPs occur in coding regions enables assessment of polymorphisms potentially directly affecting the phenotype [5]. On the other hand, as SNPs are normally bi-allelic markers, more loci are needed to obtain sufficient statistical power in certain analyses (see e.g. [6]) and allele frequencies of SNPs are usually skewed in population level analyses [7]. In theory, the limited amount of information in a single SNP locus can be compensated by increasing the number of loci screened, and several high-throughput procedures have been developed to facilitate this need (see [5,8]). Overall however, it is clear that SNPs are an important class of molecular markers for genomics research and can potentially be applied in a wide range of studies.

While the recognized benefits of SNPs have accelerated their use in studies of model organisms [9-13], the application of SNPs in genetic studies of wild species has been relatively rare. Their potential use in animal genetics has been reviewed by Vignal *et al*. [14], presenting the usefulness of SNPs in, e.g., parentage assignment, animal tagging (see also [15]) and especially in QTL mapping, but similar studies with natural organisms have only been reported recently, most likely due to a lack of suitable markers. Recently however, new SNP discovery strategies (e.g. [16]) have resulted in characterizations of SNPs in many natural populations of vertebrates to address several evolutionary, ecological and conservation issues. For example, SNPs have been applied for the identification of cryptic vole species [17], to investigate the level of genome introgression in a passerine bird hybrid zone [18,19], and to study the population genetics of wolves [20].

SNP discovery in model organisms has primarily been performed by comparing genomic information of multiple individuals in the public databases in order to identify putative polymorphic sites (e.g. [21]). This has been a useful approach for species with a wealth of nuclear sequence data available, but is not a very feasible method for the majority of non-model organisms. In species with little published sequence data available, SNP identification has been carried out by sequencing random DNA fragments (e.g. [16,22,23]), or by using a targeted gene approach where primers have been designed in conserved regions of orthologous gene sequences from closely related species to amplify less conserved regions like introns, generally termed 'comparative anchor tagged sequences' ('CATS') or the 'exon-primed intron-crossing' ('EPIC') method (e.g. [16,24-28]). Again, this latter type of SNP discovery may be challenging if the entire taxa of interest lacks published sequence data. In recent years, however, large-scale sequencing and EST projects have provided usable data for a great variety of new species and a particular increase can be seen in fish species, due in part to their potential use as models in environmental genomics [29] as well as the broad variety of species of aquaculture importance. The total number of SNPs needed to trace different strains within a species has been estimated with salmonid fish [30] and, even individual identification would be possible if the population and/or species-specific SNPs were characterized as has been conducted in wolves [20]. Furthermore, Glaubitz *et al*. [6] estimated in a simulation study that about five times more SNPs than microsatellites are needed to determine pair-wise genetic relationships.

Atlantic salmon (*Salmo salar*) has been one of the most widely studied fish species in recent decades due to its importance for aquaculture and conservation, but extensive SNP characterization studies with this salmonid species have been scarce. Recently, large-scale sequencing, EST and BAC library projects have made a large amount of data available in the public databases for *S. salar *(see [31-33]; and also [34-36]). These genomic resources have given rise to the first exploitation of EST databases for SNP discovery (unpublished data, Hayes *et al*). However, Hayes *et al*. (unpublished data) speculated that a proportion of the potential SNPs observed in *S. salar *EST sequences could in fact be a consequence of ancient duplication events in the salmonid genome, and some of the 2,507 putative EST-based SNPs found could actually be sequence differences between ancestral duplicates (i.e. paralogues) rather than true SNPs. Similarly, genome duplication has also been suggested to affect SNP identification in a recent study of Pacific salmon (*Oncorhynchus tshawytscha, O. nerka, and O. keta*) [37], where one-third of the analyzed loci were suggested to be paralogue sequence variants rather than true SNPs. Potentially sequence differences between duplicons rather than SNPs (see [38]) may emerge especially when the more highly conserved regions (i.e. exons) of the genes are used for primer design as this increases the risk of amplifying both paralogs of the same locus. Thus far, such exon-focused methods (e.g. EPIC) have been exploited in most of the SNP discovery surveys, and no study exists where the more variable, non-coding segments of the genes (e.g. introns) have been utilized to design specific primers aimed at binding to only one of the duplicated loci. This is probably due to fact that more than one sequence copy of the particular gene seldom exists in the databases and not much is known about the extent of potential duplicated genes in different species. However, intron sequences of one known duplicated gene in salmonids – growth hormone – have been used as a source of variation for phylogenetic and population studies [39-41], indicating that the divergence in introns could be sufficient for a discriminative priming strategy between putative paralogs in salmonid species.

The aim of this study was to characterize potential SNPs in the Atlantic salmon genome using gene sequence data for salmonids and other teleost species obtained from GenBank [42]. Initially, PCR primers were designed by utilizing the exonic regions of salmonid or other teleost species (EPIC). However, on observing that numerous duplicated genes had likely been amplified, a new method – termed intron-primed exon-crossing (IPEC) – was developed to circumvent this problem, whereby primers were designed in more variable intronic regions of salmonid genes. The feasibility of this new priming method to avoid amplifications of potential duplicated loci was evaluated, and the proportion of conserved duplicated loci assessed. Polymorphism was assessed by sequencing the fragments of individuals originating from 15 salmon populations spanning the species range. Furthermore, a subset of primers was tested with brown trout (*Salmo trutta*), arctic char (*Salvelinus alpinus*) and grayling (*Thymallus thymallus*) to investigate the usefulness of these loci to produce cross-species sequence data from other salmonids.

## Results

### Exon- vs. intron-primed SNP discovery strategies

Out of a total of 47 loci for which primers were designed using the EPIC strategy, only 14 (30%) primer pairs produced PCR products suitable for direct sequencing – i.e., PCR amplification resulted in a single, strong band as visualized by agarose gel electrophoresis (Table 1a). The vast majority of these (13 out of 14) were loci where primer sequences were designed using salmonid exonic sequences. However, of these 13 clear PCR products, high-quality sequence was obtained for only 4 loci, with the sequences of other loci resembling that expected if multiple sequences were present in the same reaction. For primers based on exonic sequences of non-salmonid teleosts, the proportion of loci for which a single clear PCR product was obtained was much lower (4%). However, following re-PCR of one of the multiple bands observed, high-quality sequence was obtained for a similar overall proportion of loci to that for exonic primers based on salmonid sequences (24% vs. 18%: Table 1a).

In comparison, the success rate of intron-primed exon-crossing (based on salmonid intron sequences) was considerably higher: a single clear PCR product was obtained for 21 of 22 loci (95%) and high-quality sequences were obtained for 17 of these (77%) – i.e., a success rate almost four times higher than that obtained using the EPIC approach (χ^2 ^= 7.771, d.f. = 1, *P *= 0.005). In addition, of the loci for which high-quality sequence was obtained, the proportion of loci in which polymorphism was identified was higher in IPEC-derived sequences (47% vs. 30%). This difference is even more striking when considering the proportion of polymorphic loci in the total number of loci initially tested (36% vs. 6%). In other salmonid species the proportion of loci for which sequences were successfully obtained ranged from 12% in grayling to 60% in brown trout (Table 1b). Sequences of all loci have been deposited in GenBank with the accession numbers [GenBank:DQ834872–DQ834885]. Details of the loci for which high-quality sequence data were not obtained are available on request.

### Level of genetic diversity in the gene sequences of Atlantic salmon and other species

In total, high quality sequences were obtained for 27 loci with a total of 12,911 bp. Nineteen polymorphic sites were observed in 10 loci which translates to an average of one SNP per 680 bp in the *S. salar *genome (Table 2, Additional file 2). The observed frequency is one of the lowest reported for any fish species and lower than the frequencies reported in the majority of multi-locus studies in different taxonomic groups; only some mammalian and avian studies exhibited lower estimates (Figure 2). The distribution of polymorphism among the loci was however highly skewed, with no variation observed in ~60% of loci (Table 2, Figure 3). The nucleotide diversity of individual loci ranged from 0 to 17.5 × 10^-4 ^and over all loci was 3.99 × 10^-4 ^(Table 2, Figure 3). Twelve of the polymorphic sites were located in intronic regions of verified salmonid genes whereas none occurred in the exons (Table 2). This results in the nucleotide diversity estimates of 6.7 × 10^-4 ^(1 SNP/405 bp) for introns and <1.9 × 10^-4 ^for exons (less than 1/1448 bp) respectively. As a comparison, the level of variability in transferrin, a gene suggested to have been affected by the forces of diversifying selection in salmonid fishes, was also assessed (locus sTf, Additional files 1 and 2). The nucleotide diversity of this gene was many times higher (46.0 × 10^-4^) than that observed in other genes. Furthermore, three of the five SNPs (1/77 bp) observed in this gene occurred in exonic sequences, two of which were non-synonymous.

Considering other salmonid species, the overall nucleotide diversity for *S. alpinus *was similar to *S. salar *but the estimates were about six times higher for *T. thymallus *and *S. trutta *(Table 2). Furthermore, the frequency of polymorphic sites was much higher in *T. thymallus *(1/144 bp) and *S. trutta *(1/153 bp) compared with *S. salar*, but almost identical for *S. alpinus *(1/695 bp). Contrary to *S. salar*, the transferrin gene in *S. trutta *(396 bp sequenced) exhibited no variation among the analyzed populations; instead, four SNPs were located in the exonic regions of other genes, also changing the reading frame of *tap2A *gene (Table 2).

## Discussion

The results of this study have important implications for SNP discovery in non-model species with ancestrally duplicated genomes. Exon-targeted primers using sequence data from the same or closely related taxa which have previously been used in SNP characterization studies with non-model species [16,22,26,37] were relatively unsuccessful in Atlantic salmon compared with the IPEC approach proposed here, where less conservative gene regions – i.e., introns – were the target sequences for primer design. The reduced success of the EPIC approach for SNP discovery is most likely due to the duplicated nature of the salmonid genome. This genomic duplication is suggested to have taken place in the ray-finned fish lineage after its divergence from tetrapods (reviewed in [43]) and additional, more recent polyploidization events have also been detected in the salmonid sublineage (see [44]). The subsequent re-diploidization event in the salmonid genome has generated duplicated paralogs, which may diverge from each other due to the relaxation of purifying selection in one of the copies (reviewed in [45]). Thus, assuming that diverged introns evolve even more rapidly due to lower selective pressure, the amplification of potential duplicates could be minimized by focusing on those regions for PCR primer design.

Indeed in *S. salar*, this new intron-targeted IPEC method clearly outperformed the widely used EPIC (or CATS) approach, which utilizes conserved gene regions in cross-species applications. The proportion of screened loci that revealed polymorphism in *S. salar *was around six times higher with the IPEC (36.4% polymorphic) than the EPIC (6.4%) method (Table 1a), suggesting that less effort is needed to yield the same number of SNPs than with the EPIC (or CATS) method [28,37].

Recently, special interest has focused on identifying multi-site variation after duplication from ordinary SNPs in humans [46]. Studies with several salmonid species have also speculated that some of the observed polymorphic sites could actually be variation between retained paralogs of duplicated segments rather than true SNPs ([37,38]; unpublished data, Hayes *et al*). The duplication presumably lowered the success rate of the EPIC primers, especially those designed in salmonid genes (Table 1a), but it may have a minor effect on the novel SNPs identified in this study as the IPEC method produced most of the polymorphic loci (71% in total, Table 1a–b). Therefore, this intron-focused approach should be a feasible method to avoid obtaining potential 'duplicated SNPs' when identifying novel polymorphic loci from the species bearing putative duplicated genomic fragments or even an entire duplicated genome.

The observed nucleotide diversity estimates over all loci in *S. salar *(3.99 × 10^-4^) was highly similar to that in European humans [9] and about twofold lower than that observed in a larger scale survey with human genome [4]. On the other hand, the estimations are about ten times less than reported in birds [16] or plants [47,48] and about three times less than reported in a recent study of the GH1 gene of *S. salar *[41]. The greater number of base pairs and the number of independent loci sequenced here most likely better represents the overall nucleotide diversity estimate of *S. salar *genome than that observed in a single locus [41]. A lower nucleotide diversity in *S. salar *is further supported by the fact that about 60% of all analyzed loci showed no variation (Figure 3), and the overall SNP frequency was lower than in the majority of other organisms (Figure 2 and references therein). A lower level of sequence variation could be a consequence of relatively recent colonization of *S. salar *in its present habitats in the northern hemisphere after the last glaciation about 10, 000 years ago [49] as such patterns of reduced genetic variability in areas previously glaciated areas has been observed for other northern species (e.g. [50,51]).

The SNP frequency in non-coding regions was at least threefold higher than coding regions in *S. salar*, which is to be expected due to the greater selective pressure on exons as observed in a recent human genome study [52]. Studies on disease-associated genes in humans have revealed an even higher proportion of coding SNPs, implying the effects of natural selection [53,54]. This may also explain the higher frequency of SNPs in the coding region of the transferrin gene, which plays an important role in resistance to bacterial infection in a variety of organisms and was earlier reported to be under positive selection in *S. salar *[55]. On the contrary, no polymorphisms were detected in the transferrin gene of *S. trutta*, proposing that the effects of selection may vary considerably within lineages. However this could be due to the selection of the transferrin gene region which was sequenced in this study as considerable molecular variation has been reported in the transferrin gene within European *S. trutta *populations based on electrophoretic screening [56].

The overall SNP frequencies also varied among the salmonid species examined here (between 1/144 bp in *T. thymallus *to 1/695 bp in *S. alpinus*) but were, however, within the range of the SNP frequencies for a range of multi-locus studies with different species (Figure 2 and references therein). The estimates for *S. salar *and *S. alpinus *were in congruence with a previous study on *S. salar *(unpublished data, Hayes *et al*.), whereas the frequencies for *T. thymallus *and *S. trutta *were closer to a recent study with Pacific salmon [37]. Furthermore, in *S. trutta *and *T. thymallus *the nucleotide diversities were about six times higher than in *S. salar *or *S. alpinus *(Table 2). The high level of diversity in *T. thymallus *is consistent with the deep divergence between the evolutionary lineages assessed [57]. However, the high level of diversity in *S. trutta *is more difficult to explain as all individuals analysed originate from the same evolutionary lineage (the Atlantic lineage) proposed by Bernatchez [58]. However it is important to note that no Finnish *S. trutta *samples were assessed in the study of Bernatchez [58] and thus additional diversity may be harboured in this region.

## Conclusion

Exploitation of the exponentially increasing amount of gene sequence data in public databases such as GenBank and recent EST projects is a very useful basis for identifying new polymorphic loci from the genomes of non-model organisms. Applications of SNPs have already been reported in ecological and conservation studies of natural populations [17,20,22], and these new types of markers have also been used to identify different Atlantic salmon strains [30]. However, as observed in this study, polymorphisms can be biased toward a relatively small portion of loci (Figure 3) thus increasing the effort required to identify a sufficient number of SNPs for ecological and population genetic applications. Based on a simulation study, the need for independent SNPs is fivefold that of microsatellites [6]. Furthermore, in salmonid fish the genome duplication event has been suggested to reduce SNP validation success ([37]; unpublished data, Hayes *et al*.), a result supported by this study, which may further hinder the development of a large number of independent loci. Therefore, the new IPEC approach introduced here will be a useful way to identify true SNPs for various applications in species with presumably duplicated genomes.

## Methods

### Candidate loci identification

Initially, candidate sequence fragments were extracted from GenBank using the criteria that they consisted of both exon and intron regions, the intronic regions were ~400–600 bp in length to enable a single forward or reverse sequencing read of the particular PCR product, and that there were long enough exonic sequences flanking both sides of the desired intron for PCR primer design. Then, two different EPIC approaches were used in the primer design processes: (I) primers were designed on flanking exonic sequences of *S. salar *or other salmonid genes, or (II) flanking exonic sequences of other teleost fishes were used to design oligonucleotides. In addition, when the success rate of these exon-primed primers was seen to be low, a new intron-primed exon-crossing method was introduced (hereafter called IPEC) where at least one primer was designed in the intronic regions of salmonid fish genes (Additional file 1) in an attempt to avoid amplification of potential paralogues. It should be noted that some of the primers designed in introns amplified only intronic sequences without spanning any exonic regions (6/24 in *S. salar*; Table 1a) but for the sake of uniformity all these fragments are referred as IPEC loci. Based on the criteria described above, a total of 69 PCR primer pairs (Additional file 1) predicted to amplify fragments of ~400–700 bp in total length were designed using the program Primer3 [59].

### Sampled individuals and populations

**Figure 1:**
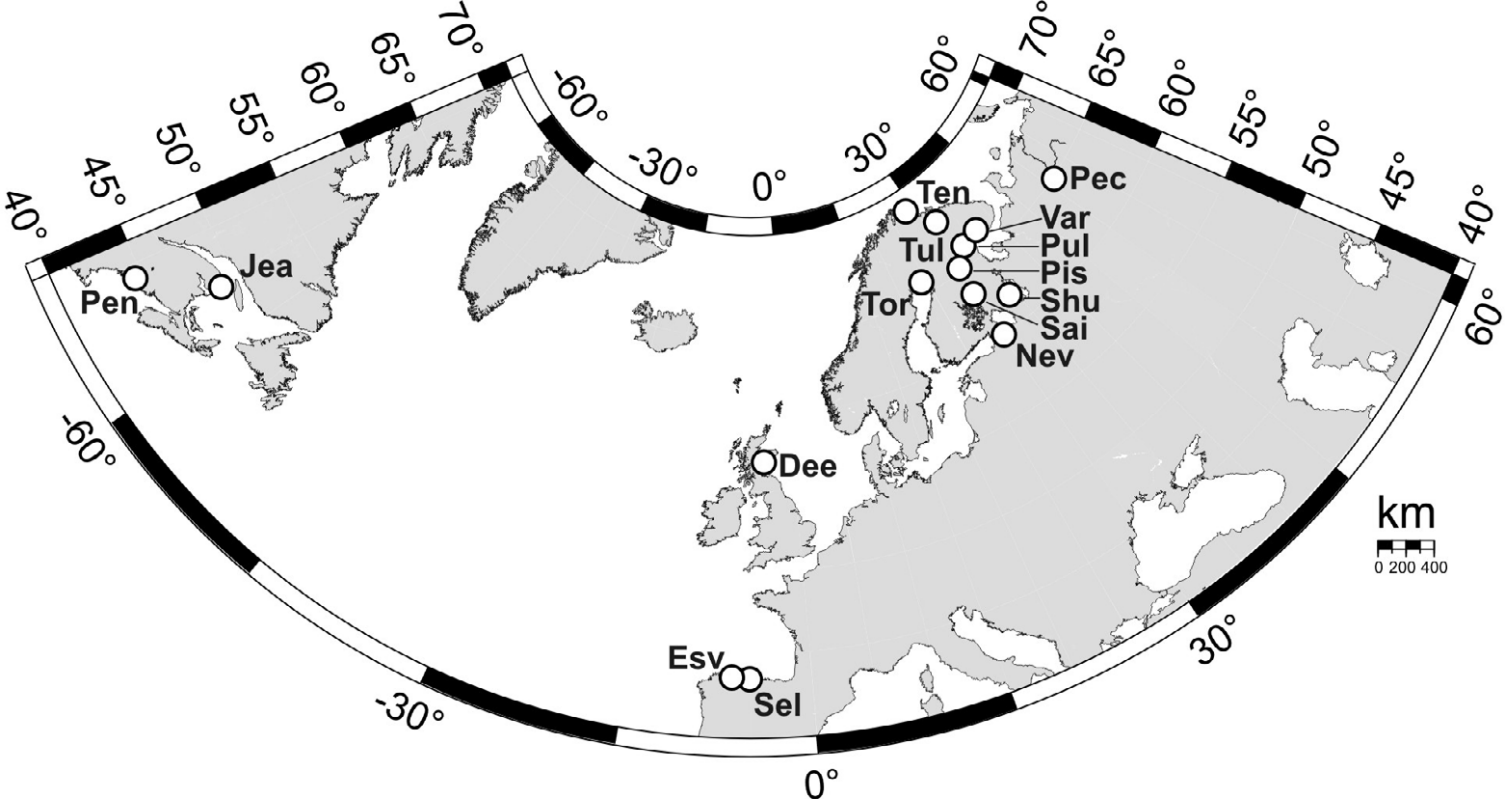
**Locations of the 15 Atlantic salmon population analyzed in this study**. One individual was sampled from each population and their abbreviations were: Penobscot, USA (Pen), St Jean, Canada (Jea); Dee River, UK (Dee); Esva River, Spain (Esv); Sella River, Spain (Sel), Tenojoki River, Finland (Ten); Tuloma River, Russia (Tul); Tornionjoki River, Finland (Tor); Pechora River, Russia (Pec); Varzuga River, Russia (Var); Pulonga River, Russia (Pul); Pistojoki River, Russia (Pis), Shuja River, Russia (Shu); Lake Saimaa, Finland (Sai) and Neva River, Russia (Nev).

**Figure 2:**
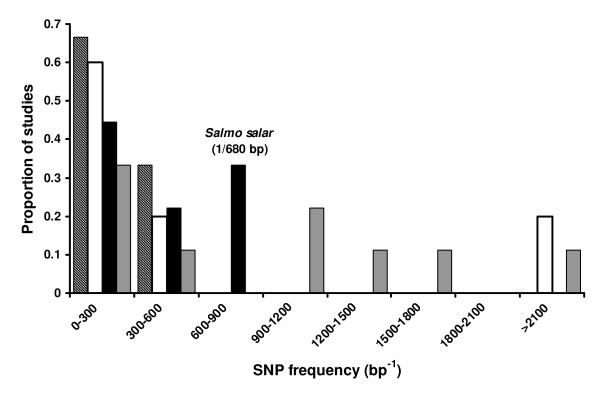
**SNP frequencies in four salmonid species observed in this study and from 25 selected multi-locus studies of various organisms**. Different species were split into four taxonomic groups: plants (n = 6, hatched bars), birds (n = 5, white bars), fish (n = 9, black bars), and mammals (n = 9, grey bars). Estimates were obtained from the following publications: [4, 9, 12, 16, 71]; [2] and references therein; [20, 23, 37, 72-74]; unpublished data, Hayes *et al*.

**Figure 3:**
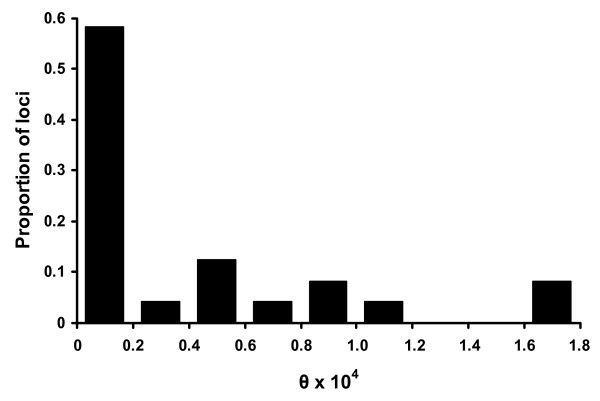
**Frequency distribution of nucleotide diversities (θ) observed in the sequences of 24 independent loci (see Table 3) investigated in Atlantic salmon**.

One *S. salar *individual per population from each of 15 populations covering a wide range of the species' distribution in Europe and North America were assessed for polymorphism (Figure 1). Of these, Rivers Pistojoki and Shuja and Lake Saimaa exhibit a non-anadromous migration behaviour, whereas all others were anadromous populations. Furthermore, the Lake Saimaa and River Neva samples were of hatchery origin. Different subsets of primers were also tested with five other salmonid (*S. trutta*, *S. alpinus *and *T. thymallus*) populations (one individual per population) around Europe to investigate the cross-species amplification success of these loci: *S. trutta *samples (n = 5) were from Poland, Scotland and three locations in Finland; *S. alpinus *samples (n = 5) were from Russia, Norway, Scotland and two locations in Finland; and *T. thymallus *samples (n = 5) were from Norway, Russia, Slovenia and two locations in Finland. Genomic DNA was extracted using ethanol-preserved tissue samples and either a salt extraction protocol [60] or a silica-based method [61].

### Amplification and sequencing of the loci

Details of all primers used in this study are presented in Additional file 1. PCR amplifications were carried out in a total volume of 20 μl as outlined in Ryynänen and Primmer [62] and using the primers and annealing temperatures outlined in Additional file 1. In general, all PCR programs were first optimized using the 'touchdown' PCR protocol described in [63], except that the extension step was 45 s at 72°C. More specific PCR programs were then used for those loci which produced clear PCR products in the initial amplifications.

As PCR amplifications with primers designed in sequences of non-salmonid species generated multiple fragments in most of the loci, re-PCR amplifications were performed for PCR bands extracted from agarose gels (see Additional file 1) to obtain a single PCR product for sequencing. The initial PCR products were visualized on 1–2% agarose gels stained with ethidium bromide, and the strongest band was selected to represent the amplicon of the particular locus. A small piece of gel including the desired PCR product was pierced with a plastic pipette tip and, to elute the DNA fragments, the gel piece was dissolved in 50 μl of H_2_O and incubated for at least one hour at room temperature. The re-PCR amplification was then performed with the same primers and protocol as before, except for reducing the number of PCR cycles to 30 and using 1–2 μl of the eluted PCR fragment as a template.

The PCR products were cleaned with GFX™ DNA purification columns (Amersham Biosciences) or Montage^® ^PCRμ96 Plates (Millipore) to remove unincorporated nucleotides and primers before direct sequencing. The PCR products were then cycle sequenced in both directions using the BigDye Terminator Cycle Sequencing Ready Reaction Kit 1.0 premix (PE Biosystems) as recommended by the manufacturer, using one of the original PCR primers in turn (Additional file 1) as sequencing primers. After sequencing, the products were purified using Sephadex spin columns (Amersham Biosciences) or Montage^® ^SEQ_96 _Plates (Millipore), and electrophoresed with an ABI 377 automated sequencer (PE Biosystems) following the manufacturer's recommendations.

### Data analysis

Sequenced loci from different populations were base-called and aligned using the 'SNP pipeline' [21] – accessible from SNP analysis [64] web server- which employs the Phred/Phrap/PolyPhred series of base-calling, alignment and SNP identification programs [65-67]. All putative SNP sites, either heterozygous or homozygous, were also inspected and evaluated manually and only approved as 'true SNPs' if they met at least one of the following criteria: high-quality sequences (phred score ≥ 20) of the rarer nucleotide variant obtained (i) in one or more individuals in both directions (69.2% of the SNPs observed), (ii) in one direction for at least two individuals (23.1%), or (iii) in one individual in one direction in a region of high sequence quality (7.7%). The classification of validated SNPs in other salmonids was 38.7%, 22.6% and 38.7% respectively. Low-quality single-read sequence regions were excluded from all analyses. Candidate sequences obtained with the primers designed in non-salmonid fish sequences were subjected to a Blast homology search [68] against GenBank [42] and the Atlantic Salmon Gene Index [36] to reveal putative homologous genes from the salmon genome.

Nucleotide diversities for the successfully sequenced PCR fragments were estimated using the formula 'theta' = *K*/[L * [1^-1 ^+ 2^-1 ^+ 3^-1 ^+ ... + (*n*-1)^-1^]], where *K *is the number of observed polymorphic sites, *L *is the total length of the sequence (in bp) and *n *is the total number of chromosomes screened. The formula corrects for different sequence lengths and variation in the number of gene copies analysed [69,70]. The overall nucleotide diversity estimate was calculated by averaging the number of loci over all screened (ranged from 8 to 30; Table 2). As the analysed transferrin locus is reported to be under selective constraints in salmonids [55], it was excluded in the estimation of the overall nucleotide diversity.

## Authors' contributions

HJR carried out the molecular genetic analyses, performed the data analysis and drafted the manuscript. CRP conceived the study, participated in its design and helped to draft the manuscript. Both authors read and approved the final manuscript.

## Supplementary Material

Additional file 1 Details of loci, derived from different teleost fish species, tested in the initial SNP screening phase. Indicators i-iv refer to fragments amplified from the same gene. Data provides detailed information of the different loci tested in the initial SNP screening phase in this study. Click here for file

Additional file 2 Details of the polymorphic sites detected at the sequenced fragments in fifteen Atlantic salmon populations. Overlapping fragments from the same gene have been combined as one index. Population abbreviations are given in Figure 1. Details of these SNPs have been included the sequences submitted to GenBank with the accession numbers [GenBank:DQ834848–DQ834872]. Data provides detailed information of the observed polymorphic sites in the analyzed loci. Click here for file

## Figures and Tables

**Table 1a:** Summary of the success of candidate SNP loci identification with different priming approaches in (a) Atlantic salmon.

	PCR primer design strategy			
		
Process description	EPIC I ^a^	EPIC II ^b^	IPEC ^c^	Total
*S. salar*				
No. loci tested	22	25	22	69
No. loci producing clear PCR product	13 ^d^	1 ^e^	21	36
No. loci successfully sequenced	4	6	17	27
No. polymorphic loci	0	3	8	11

a – primers in exons of salmonid genes

b – primers in exons of other teleost genes

c – at least one primer in intron regions of salmonid genes

d – in five loci two distinct PCR bands were observed and four loci produced a smear

e – most of the primers produced several PCR bands and thus re-amplifications were needed (see Methods)

**Table 1b:** Summary of the success of candidate SNP loci identification with different priming approaches in (b) other salmonids.

	PCR primer design strategy			
		
Process description	EPIC I ^a^	EPIC II ^b^	IPEC ^c^	Total
*T. thymallus*				
No. loci tested	22	-	20	42
No. loci producing clear PCR product	11 ^d^		4	16
No. loci successfully sequenced	4	-	2	5
No. polymorphic loci	3	-	2	5
*S. trutta*				
No. loci tested	-	-	10	10
No. loci producing clear PCR product	-	-	8	8
No. loci successfully sequenced	-	-	6	6
No. polymorphic loci	-	-	4	4
*S. alpinus*				
No. loci tested	-	-	10	10
No. loci producing clear PCR product	-	-	5	5
No. loci successfully sequenced	-	-	3	3
No. polymorphic loci	-	-	1	1

a – primers in exons of salmonid genes

b – primers in exons of other teleost genes

c – at least one primer in intron regions of salmonid genes

d – in five loci two distinct PCR bands were observed and six loci either produced a smear or no amplification was observed

**Table 2:** Details of the sequenced fragments and polymorphisms indices found in analysed salmonid fish populations. Overlapping loci from the same gene have been combined as one index. Details of these SNPs have been included the sequences submitted to GenBank with the accession numbers [GenBank:DQ834848–DQ834885].

		Focal species				
		
Locus	Source species ^a^	Sequence length (bp)	No. individuals screened	Intronic/exonic region (bp)	No. SNPs and/or indels	Theta (x 10^4^)
*S. salar*						
U2A' (ii and iv)	*Ssa*	609	14	529/80	0	-
SS-II (i and ii)	*Ssa*	540	15	403/137	0	-
HMG-1 (ii)	*Omy*	505	15	475/30	0	-
TGF-beta (i and ii)	*Omy*	791	15	489/302	1	3.2
HMG-1 (iii)	*Omy*	540	15	394/146	0	-
Ran1 (iii)	*Ssa*	501	10	416/85	3	16.6
sGnRH (ii)	*Ssa*	562	15	481/81	0	-
GDF-8	*Ssa*	497	11	497/0	2	11.0
FGF6 (ii)	*Omy*	557	11	483/74	2	9.9
Vtg (ii)	*Omy*	447	15	277/170	0	-
ras-1(ii)	*Omy*	536	5	536/0	0	-
tnfa (ii)	*Omy*	522	13	462/60	0	-
IL-1 beta 2	*Omy*	399	10	399/0	1	7.1
RAG-1	*Omy*	563	15	563/0	0	-
c-myc	*Omy*	578	15	328/250	1	4.4
D(2)R	*Omy*	483	14	483/0	0	-
IgMh	*Sal*	518	15	518/0	2	9.7
rps24	*Tru*	776 ^c^	11	unknown	5	17.5
CCBL1	*Tru*	298 ^d^	4	unknown	0	-
epn ^b^	*Cca*	359 ^e^	5	unknown	0	-
epn(2) ^b^	*Cca*	297 ^f^	9	unknown	0	-
ssg(i)	*Dre*	470 ^g^	15	unknown	1	5.4
EF1a	*Dre*	729 ^h^	5	unknown	1	4.8
hox	*Dre*	834 ^h^	9	unknown	0	-
Total		12 911	11.7 ^i^	7 700/1 448 ^j^	19	3.99
*S. trutta*						
SS-II (ii)	*Ssa*	385	5	353/32	1	9.2
tap2A (ii)	*Ssa*	367	5	238/129	6 ^k^	57.8
HMG-1 (iii)	*Omy*	540	4	394/146	0	-
TGF-beta (ii)	*Omy*	487	5	336/151	2	14.5
tnfa (ii)	*Omy*	514	4	454/60	6 ^l^	45.0
Total		2293	4.6 ^i^	1775/518	15	24.1
*T. thymallus*						
SS-II (i)	*Ssa*	525	5	416/109	5	33.7
U2A' (ii)	*Ssa*	373	5	327/46	4	37.9
TGF-beta (i and ii)	*Omy*	743	5	441/302	3	14.3
RAG-1	*Omy*	376	5	376/0	2	18.8
Total		2017	5 ^i^	1560/457	14	24.5
*S. alpinus*						
SS-II (ii)	*Ssa*	354	5	322/32	0	-
HMG-1 (iii)	*Omy*	524	5	378/146	2	13.5
tnfa (ii)	*Omy*	512	5	452/60	0	-
total		1390	5 ^i^	1152/238	2	5.1

a – *Cca, Cyprinus carpio; Dre, Danio rerio; Omy, Oncorhynchus mykiss; Sal, Salvelinus alpinus; Ssa, Salmo salar; Tru, Takifugu rubripes*

b – two separate PCR fragments extracted from the agarose gel were sequenced using the same primer pair

c – 92% (346/376 bp) homology to * S. salar *zonadhesin-like gene, promoter region [GenBank:AY785950]

d – 94% (228/242 bp) homology to * O. mykiss *multi tissue cDNA clone [GenBank:BX076795]

e – 98% (195/198 bp) homology to * S. salar *white muscle cDNA library clone [GenBank:CK899537]

f – 93% (230/245 bp) homology to * S. salar *mixed tissue cDNA clone [GenBank:CA055131]

g – 95% (334/350 bp) homology to * S. salar *kidney cDNA library clone [GenBank:CK886988]

h – no homologous sequences found after Blast search

i – an average number of individuals screened over all loci

j – the sum of intronic/exonic regions do not include 'unknown' sequences in * S. salar*

k – three SNPs occurred as non-synonymous form in exonic regions

l – one polymorphic site was located in synonymous site of exonic region
