# Supplementary material for: Single nucleotide polymorphism (SNP) discovery in duplicated genomes: intron-primed exon-crossing (IPEC) as a strategy for avoiding amplification of duplicated loci in Atlantic salmon (Salmo salar) and other salmonid fishes
Source: BMC Genomics. 2006 Jul 27;7:192. doi: 10.1186/1471-2164-7-192 (PMC1557852; doi:10.1186/1471-2164-7-192)
Supplement: Additional file 1 — Details of loci, derived from different teleost fish species, tested in the initial SNP screening phase. Indicators i-iv refer to fragments amplified from the same gene. Data provides detailed information of the different loci tested in the initial SNP screening phase in this study. [file 1471-2164-7-192-S1.doc]

**Additional file 1.** Details of loci, derived from different teleost fish species, tested in the initial SNP screening phase. Indicators i-iv refer to fragments amplified from the same gene.

| Locus ^a^ | Full gene name | Accession (GenBank no.) | Primer location | Oligonucleotide primers sequences (5’-3’) | Annealing temp (°C) | Sequenced species ^b^ |
| --- | --- | --- | --- | --- | --- | --- |
| *Salmo salar* |  |  |  |  |  |  |
| U2A' (i) ^1^ | U2 snRNP-specific A' protein | AJ004823 | exon5 exon6 | F: AGCACTACAGGCTCTATGTCATCA R: ATATCCTTTGCAAGCTGAGCAC | 60-50 ^c^ | - |
| U2A' (ii) ^1^ | as above | AJ004823 | exon6  exon7 | F: GAGAAAATGTTCAAGGGCAAAC  R: AGACGGTCCCATCTTCTTCTTCT | 60 | *Sss, Tth* |
| U2A' (iii) ^1^ | as above | AJ004823 | exon7  exon8 | F: CAGCAGCCTGAGAAGAAGAAGAT  R: GGGTCAGGGCAGTGTGTAGAT | 60-50 ^c^ | - |
| U2A' (iv) ^2^ | as above | AJ004823 | intron5  intron6 | F: CAATGATAAAGAAGCATGCAGAAT  R: CAGCAGAGTTAGAGAGAACATGGA | 44 | *Ssa*, |
| Ran1 (i) ^1^ | Ran1 | AJ012477 | exon2  exon3 | F: AAGACCACTTTCGTCAAAAGACAT  R: GTATTTGATGGCCCCTCTGTT | 60-50 ^c^ | - |
| Ran1 (ii) ^1^ | as above | AJ012477 | exon3  exon4 | F: CATCAAATACAATGTGTGGGACA  R: GGTACGTTCTTGTAGGTGACTCG | 60-50 ^c^ | - |
| Ran1 (iii) ^2^ | as above | AJ012477 | intron1  intron2 | F: GAGGGTTTTTCCTTGTATCTTTCA  R: GAGGTAGCGAGAAATAAGGAAGTG | 44 | *Ssa* |
| sCP ^1^ | cardiac peptide hormone | AJ006421 | exon1  exon2 | F: AGACCATACCCTGATAGCGACTT  R: CAGCCAAGTTCTCCTCGAATC | 60-50 ^c^ | - |
| tap2A (i) ^1^ | transport-associated protein | Z83325 | exon4  exon5 | F: GCAAGGCGTCTATGACAACTATTA  R: CTTAGAGAGCCTCTGTGGAACAGT | 60-50 ^c^ | - |
| tap2A (ii) ^2^ | as above | Z83325 | intron5  intron6 | F: CCTTAGGCTCAGAAGAGTTGAATG  R: CTGGAGAGGGCAGATGTATTAT | 60-50 ^c^ | *Str* |
| beta-globin ^1^ | beta-globin | X97287 | exon1  exon3 | F: ATATCAATGAGGTCGGACCACTG  R: ATGACAATTGTGAGGACGTCAG | 60-50 ^c^ | - |
| sTf (i) ^1^ | transferrin | L26909 | exon1  exon3 | F: AGACTTCGAGAACATGAAACTGC  R: ATGCATGTACCATGTGCGTAAC | 60-50 ^c^ | - |
| sTf (ii) ^2^ | as above | L26909 | intron1  intron2 | F: AAGTGGGCTTATACATGCAAAGAT  R: ATGCATGTACCATGTGCGTAAC | 44 | *Ssa, Str* |
| sGnRH (i) ^1^ | prepro-gonadotropin releasing hormone | X74957 | exon3  exon4 | F: TCTTCCTGAGGAGACAAGTGC  R: ACAGTTCTTTATTTATGGGGCATC | 60-50 ^c^ | - |
| sGnRH (ii) ^2^ | as above | X74957 | intron2  intron3 | F: CATACTGTGCATGTAATTGTCCTTT  R: GGGAAATCAAAGTGTTAACTTCAGA | 45 | *Ssa* |
| SS-II (i) ^1^ | ependymin | M93699 | exon1  exon3 | F: CTCCACAAGAAGATGCAGGACT  R: AGTCGTAGCTGTAATGTCCAGTTG | 50 | *Ssa, Tth* |
| SS-II (ii) ^2^ | as above | M93699 | intron1  intron2 | F: CACTGCAGTAAGGATTATCTGCTT  R: GAGGAAGAGCGAGGAAGATATAAAG | 50 | *Ssa, Str, Sal* |
| alpha-globin ^2^ | alpha globin | X97284 | intron2  intron1 | F: TTAAATAATAACACGATAATAGGT  R: GAGAATGAAATATTGGAATAAG | 60-50 ^c^ | - |
| GDF-8 ^2^ | myostatin | AJ316006 | intron2  intron2 | F: TTTGCTCTCATGTGCTCATATTTT  R: GTCCCCACAAGAATAGTAACCAAC | 42 | *Ssa* |
| *Oncorhynchus mykiss* |  |  |  |  |  |  |
| HMG-1 (i) ^1^ | testis high-mobility-group-1 protein | L32859 | exon2 exon3 | F: AGAGTTCTCCAAGAAGTGCTCTGA R: ATCTCCCTCTCATATCGCACCT | 60-50 ^c^ | - |
| HMG-1 (ii) ^1^ | as above | L32859 | exon4  exon5 | F: ACAAGGTACCCTATGAGAAGAAGG  R: GTACGCAGTGATGTCCTGAGAATA | 60 | *Ssa* |
| HMG-1 (iii) ^2^ | as above | L32859 | inron2  intron3 | F: TCAATCCCTTGATAGTTGTCTTTG  R: GGGACACCCTGATTTTAATTGTAG | 47 | *Ssa, Str, Sal* |
| FGF6 (i) ^1^ | fibroblast growth factor 6-related protein | Y16850 | exon1  exon2 | F: ACGGGAGGATAAATGGTGTACATA  R: CGCTCTACCGTTGAGATCTCTATTA | 60-50 ^c^ | - |
| FGF6 (ii) ^2^ | as above | Y16850 | intron1  intron2 | F: CAACCTATTTTTACACTGGCTCCT  R: ACCAAATAGCCTACCATTCCATTA | 45 | *Ssa* |
| IL-1 beta ^1^ | interleukin-1 beta | AJ004821 | exon5  exon6 | F: GATCCAACCTCTACCTGTCCTG  R: ATGGACTTCAGCTGATCCTTGT | 60-50 ^c^ | - |
| Vtg (i) ^1^ | vitellogenin | AJ011695 | exon1  exon4 | F: GACTCTAGCCCTTGTGGGTAAGTA  R: AAGATCTCAGGGTTCACAAGCTAC | 60-50 ^c^ | - |
| Vtg (ii) ^2^ | as above | AJ011695 | intron1  intron3 | F: TTTTTGTCTTATTCTAGTGCAGAT  R: GATATTGTTTGTGTGGGAACTTCA | 42 | *Ssa* |
| ras-1 (i) ^1^ | ras-1 protein | M73690 | exon1  exon2 | F: GAACCACTTTGTGGATGAATATGA  R: CTGAAGGAGGGAGAGAGAAAGAG | 60-50 ^c^ | - |
| ras-1 (ii) ^2^ | as above | M73690 | intron1  intron1 | F: ACACCCTTGTCTCTTACTTCAGCA  R: AAGGAGGGAGAGAGAAAGAGAGG | 45 | *Ssa* |
| IgMh ^1^ | immunoglobulin M constant region | X83372 | exon3  exon4 | F: GGACTTGGTAAAGAAAGCCTACAA  R: ATCACTAGTTTGTTCTGCTGGAG | 60-50 ^c^ | - |
| TGF-beta (i) ^1^ | transforming growth factor-beta | AJ007836 | exon3  exon5 | F: GAGGAATTCCGCTTCAAAATATC  R: AAGTCAATGTAAAGTTTTCGCACA | 50 | *Ssa, Tth* |
| TGF-beta (ii) ^2^ | as above | AJ007836 | intron4  intron5 | F: GTGAGACTATCCTTTATTCCAACG  R: TGTGGTTGGGTAAACAACAGTAGA | 50 | *Ssa, Str, Tth* |
| tnfa (i) ^1^ | tumor necrosis factor alpha | AJ278085 | exon3  exon4 | F: CCACCATACATTGAAGCAGATTG  R: CAGCGGAAAGATTAGGATTGTATT | 60-50 ^c^ | - |
| tnfa (ii) ^2^ | as above | AJ278085 | intron2  intron3 | F: CTTTTCATTGAATTGCTTCTCACT  R: GCAGTAGAAGGAAGAAAGGTTCAT | 44 | *Ssa, Str, Sal* |
| IL-1 beta 2 ^2^ | interleukin-1 beta 2 precursor | AJ245925 | intron5  intron5 | F: ATAATTGTCTGTTGAGGCTGGAGT  R: TGGAGAGAGAGAGAGGGAGAGATA | 56 | *Ssa* |
| RAG-1 ^2^ | recombination activating protein | U15663 | intron1  intron1 | F: TAATCACAGGATTTGAGCCACTTA  R: GAGAGTGATGGAGAGAAACAAAGG | 55 | *Ssa, Tth* |
| c-myc ^2^ | Cellular myc proto-oncogene | M13048 | exon1  intron1 | F: AGTTAGAGAAAGTGGTGTCTGAA  R: CTGCAATGATATTGAATAAAACTA | 50 | *Ssa* |
| D(2)R ^2^ | dopamine D2 receptor | AF479756 | intron5  intron5 | F: TCATAGTGTTTTTGGAGAATGGAA  R: AGTGATTAGGACTATTTGCCTTGC | 55 | *Ssa* |
| ER ^2^ | estrogen receptor | L39955 | intron1  intron1 | F: CCATATTTGCACATAAATCCACTG  R: GGCATTCACTAGGTTCTCTCTCTC | 60-50 ^c^ | *Ssa* |
| *Oncorhynchus tschawytscha* |  |  |  |  |  |  |
| isl-1 ^2^ | insulin enhancer binding protein | X64885 | N/A ^e^ N/A | F: GGCAAGACATACTGCAAAAGAGAT R: GTTTGCACATTTAATCCCAAATAA | 60-50 ^c^ | - |
| *Oncorhynchus keta* |  |  |  |  |  |  |
| insulin ^1^ | preproinsulin | X13559 | exon1 exon2 | F: CCTCAGCATTTTTGCTATTCTTCT R: ACCAGGTGAGAGCCACACAG | 60-50 ^c^ | - |
| SL ^2^ | somatolactin | D10638 | intron3  intron4 | F: TATCGGTGACTTTACTGTTGAAGC  R: AGTACACAGCAAGGACAAAACAGA | 60-50 ^c^ | - |
| IGF-II ^2^ | insulin-like growth factor II | X97225 | intron1  intron1 | F: GTGTGTGTTTGTTTTGGTGAGAAG  R: GCGTCTTGAAATGTATCCAATATG | 60-50 ^c^ | - |
| *Salvelinus alpinus* |  |  |  |  |  |  |
| IgMh ^2^ | immunoglobulin M constant region | X83373 | intron1 intron1 | F: ACCTTAGGGCAAATTAAACAATCA R: CAAAAACCAGAGATTGCAAAGTTA | 42 | *Ssa* |
| *Takifugu rubripes* |  |  |  |  |  |  |
| rps24 ^1^ | ribosomal protein S24 | AJ001398 | exon3 exon4 | F: CAAGAAAAATGAGCCCAAACACAGA R: CGTTCTTTGCGTTGTTTCCTAGAAG | 48 ^d^ | *Ssa* |
| Scn8a ^1^ | voltage-gated sodium channel alpha subunit | U97673 | exon18N  exon18A | F: GGAGAAACATTGTTAGAGGGGCAAG  R: AGCGCATTAGCTACAAGGCTGACTA | 55-45 ^c^ | - |
| CCBL1 ^1^ | cysteine conjugate beta-lyase | Y17462 | exon1  exon2 | F: CATGTCAAGACGAATTCATGCAAAC  R: TTTGCTGTAGGCTGCTGCTAATTGT | 46 | *Ssa* |
| PTHrP ^1^ | parathyroid hormone-related protein | AJ249391 | exon1  exon2 | F: AGTGACTCTTGATGGGAAACCAGTG  R: GTGGACCTCTTCCAGCAGCTTGT | 55-45 ^c^ | - |
| hiss ^1^ | histidyl-tRNA synthetase | Z54243 | exon4  exon5 | F: TTGTCACTCAGATATGACCTCACT  R: CGTTTTATGTTGGTTATCTTGTTC | 55-45 ^c^ | - |
| sna1 ^1^ | snail1 protein | AJ238206 | exon2  exon3 | F: CCACATACGCACACACACAG  R: CGCACTGGTACTTTTTCACTT | 55-45 ^c^ | - |
| PTPRc ^1^ | protein tyrosine phosphatase | AJ243430 | exon1  exon2 | F: CTACAAGAGAAAGATTGCTGATGA  R: TTCACGGTGTACTTGGAGAATA | 55-45 ^c^ | - |
| top1-beta ^1^ | topoisomerase I beta | AL035358 | exon1  exon2 | F: ACACACACTTCTCATTTTTGTTTT  R: AGCTGGGTGTCACAGACTTAC | 55-45 ^c^ | - |
| *Carassius auratus* |  |  |  |  |  |  |
| gfGTHII-beta ^1^ | gonadotropin II beta subunit | AB015596 | exon1 exon2 | F: GGTTTCATGGCACTATATAAGTCT R: ATGTTGGTAGACAGTGGAAAAT | 55-45 ^c^ | - |
| MHC I ^1^ | major histo-compatibility complex | L10418 | exon2  exon3 | F: GACTGGATCTCAACGTTTAATAAC  R: TCTGATGTTCATCTCAATATCTCT | 55-45 ^c^ | - |
| gfTSHbeta ^1^ | thyrotropin beta subunit | AB020320 | exon1  exon2 | F: GTCTATATAAGAACAGCAGCCTTT  R: CAACAACATACACAGGAGACATA | 55-45 ^c^ | - |
| *Cyprinus carpio* |  |  |  |  |  |  |
| TLAI alpha-1 ^1^ | cell surface glycoprotein alpha-1 | M37107 | exon2 exon3 | F: ATTCCTCAAGAACTGCCTGGACTG R: GCAAACACATGAACATCTGGTGAAT | 55-45 ^c^ | - |
| epn ^1^ | ependymin precursor | U00432 | exon2  exon3 | F: ATTTCTTTTTCAGATTCGCCACCAT  R: CATAACTGAACTCTCCAGATGCAAGG | 43 ^d^ | *Ssa* |
| ZP3 ^1^ | egg membrane protein | L41637 | exon4  exon5 | F: AGATATTCCTTCATTGAGAATCA  R: GATCTTGTCTTCCTGGGTTC | 55-45 ^c^ | - |
| gamma m3 ^1^ | gamma m3 crystallin | X55946 | exon1  exon3 | F: TATAAAGTATAAAAGGCAGCAGTC  R: TCTCGTAGATCCTCATTCTGTAG | 55-45 ^c^ | - |
| *Danio rerio* |  |  |  |  |  |  |
| Zpeg1 ^1^ | alpha/beta hydroxylase | AB042295 | exon10 exon11 | F: AACCAGAGGCTGAAACACAGAGAAC R: CAGAAACTGCGGATGTGGATTAACT | 55-45 ^c^ | - |
| apoE ^1^ | apolipoprotein E precursor protein | AJ236882 | exon4  exon5 | F: CACCTACACACGCAAACTGAAGAAA  R: GGGACTGCATCTCACTCATGTAGGT | 55-45 ^c^ | - |
| ssg (i) ^1^ | spermine synthase | AJ009633 | exon8  exon10 | F: TGACTTGACAGCAGTCCCTATTTCC  R: CGCATCAGTCAGGTTCACACAATTA | 43 ^d^ | *Ssa* |
| ssg (ii) ^1^ | as above | AJ009633 | exon2  exon3 | F: TATATTTCAAGAGCAGGAAATGAC  R: ATGGGAGTGCATTCGTAAGAT | 55-45 ^c^ | - |
| FGF3 ^1^ | fibroblast growth factor 3 | Z48714 | exon2  exon3 | F: GTTTTCGGGAAGATACCTGGCTATG  R: CTCCAGAAACTCACACTCTCGGTTG | 55-45 ^c^ | - |
| Epd ^1^ | ependymin beta and gamma chains | M89643 | exon2  exon3 | F: GTTTCTTTTACAGATTCACCACAG  R: AGTCATAACTGAACTCTCCAGATG | 55-45 ^c^ | - |
| beta 2m ^1^ | beta-2-microglobulin | L05384 | exon1  exon2 | F: ACTCATCACTTTTGCACTTCTTT  R: GGAAAATGACTGTACACATGAACT | 55-45 ^c^ | - |
| egr1 ^1^ | zinc finger trans-criptional regulator | U12895 | exon1  exon2 | F: GTTTGATCACCTTGCTGGAG  R: ATGGAAATTTCTGAAAGCGTAT | 55-45 ^c^ | - |
| EF1a ^1^ | translation elongation factor 1 alpha | L47669 | exon1  exon2 | F: TTTTTCTTCCTCTTTCTGTTACCT  R: CCTTTCCCATGATTGATAAGTT | 45 ^d^ | *Ssa* |
| hox ^1^ | homeobox protein | X68324 | exon1  exon2 | F: AGATATATCCGTGGATGACAAAG  R: GTGACCTTTTACCGTCAGATTC | 45 ^d^ | *Ssa* |

a – species names indicate the species of origin of the sequences. Superscript number refers to the primer design method for the particular locus: 1 – ’EPIC’, 2 – ’IPEC’

b – *Ssa, Salmo salar; Str, Salmo trutta; Sal, Salvelinus alpinus; Tth, Thymallus thymallus*

c – a touchdown PCR protocol was applied (see Methods)

d – a re-PCR amplification was performed (see Methods)

e – primers in mRNA sequence
