# Supplementary material for: Single nucleotide polymorphism (SNP) discovery in duplicated genomes: intron-primed exon-crossing (IPEC) as a strategy for avoiding amplification of duplicated loci in Atlantic salmon (Salmo salar) and other salmonid fishes
Source: BMC Genomics. 2006 Jul 27;7:192. doi: 10.1186/1471-2164-7-192 (PMC1557852; doi:10.1186/1471-2164-7-192)
Supplement: Additional file 2 — Details of the polymorphic sites detected at the sequenced fragments in fifteen Atlantic salmon populations. Overlapping fragments from the same gene have been combined as one index. Population abbreviations are given in Figure 1. Details of these SNPs have been included the sequences submitted to GenBank with the accession numbers [GenBank:DQ834848–DQ834872]. Data provides detailed information of the observed polymorphic sites in the analyzed loci. [file 1471-2164-7-192-S2.doc]

**Additional file 2.** Details of the polymorphic sites detected at the sequenced fragments in fifteen Atlantic salmon populations. Population abbreviations are given in Figure 1. Details of these SNPs have been included the sequences submitted to GenBank with the accession numbers [GenBank:DQ834848-DQ834872].

|  | Population | | |  |  |  |  |  |  |  |  |  |  |  |  |  |  |  |
| --- | --- | --- | --- | --- | --- | --- | --- | --- | --- | --- | --- | --- | --- | --- | --- | --- | --- | --- |
| Locus and position ^a^ | Pen | Jea | Tor | Ten | Pec | Dee | Sai | Sel | Esv | Shu | Tul | Pul | Nev | Var | Pis |  | Overall allele frequencies | *H*_O_ |
| TGF-beta (i and ii) | |  |  |  |  |  |  |  |  |  |  |  |  |  |  |  |  |  |
| 347 | A/A | A/A | A/A | A/A | A/A | −/− | A/A | A/A | A/A | A/A | A/A | A/A | A/A | A/A | A/A |  | A = 0.93; − = 0.07 | 0 |
| Ran1 (iii) |  |  |  |  |  |  |  |  |  |  |  |  |  |  |  |  |  |  |
| 472 | G/G | G/G | na | T/T | T/T | T/T | G/G | na | T/T | na | na | T/T | T/T | T/T | T/T |  | T = 0.73; G = 0.27 | 0 |
| 497 | C/T | C/T | na | T/T | T/T | T/T | C/C | na | na | na | na | T/T | T/T | T/T | T/T |  | T = 0.8; C = 0.2 | 0.2 |
| 498 | A/T | A/T | na | A/A | A/A | A/A | T/T | na | na | na | na | A/A | A/A | A/A | A/A |  | A = 0.8; T = 0.2 | 0.2 |
| GDF-8 |  |  |  |  |  |  |  |  |  |  |  |  |  |  |  |  |  |  |
| 67 | A/A | A/A | C/C | C/C | C/C | C/C | C/C | na | na | na | na | C/C | C/C | C/C | C/C |  | C = 0.82; A = 0.12 | 0 |
| 293 | G/G | G/G | C/C | C/C | C/C | C/C | C/C | na | na | na | na | C/C | C/C | C/C | C/C |  | C = 0.82; G = 0.12 | 0 |
| FGF6 (ii) |  |  |  |  |  |  |  |  |  |  |  |  |  |  |  |  |  |  |
| 402 | C/C | C/C | C/C | C/C | na | A/A | A/A | A/C | A/C | C/C | A/C | A/C | C/C | na | C/C |  | C = 0.77; A = 0.23 | 0.31 |
| 510 | T/T | na | G/G | T/T | na | G/G | G/G | G/G | G/T | G/G | na | na | G/G | na | na |  | G = 0.72; T = 0.28 | 0.11 |
| IL-1 beta 2 |  |  |  |  |  |  |  |  |  |  |  |  |  |  |  |  |  |  |
| 85 | na | T/T | C/C | C/T | C/T | C/T | T/T | na | T/T | na | C/T | T/T | C/C | na | na |  | T = 0.60; C = 0.40 | 0.40 |
| c-myc |  |  |  |  |  |  |  |  |  |  |  |  |  |  |  |  |  |  |
| 486 | −/− | TT/− | TT/ TT | TT/ TT | TT/ TT | TT/ TT | TT/ TT | TT/ TT | TT/ TT | TT/ TT | TT/ TT | TT/ TT | TT/ TT | TT/ TT | TT/ TT |  | TT = 0.90; − = 0.10 | 0.07 |
| IgMh |  |  |  |  |  |  |  |  |  |  |  |  |  |  |  |  |  |  |
| 423 | T/T | T/T | T/T | C/C | T/T | T/T | T/T | T/T | T/T | T/T | T/T | T/T | T/T | T/T | T/T |  | T = 0.93; C = 0.07 | 0 |
| 424 | C/C | C/C | C/C | T/T | C/C | C/C | C/C | C/C | C/C | C/C | C/C | C/C | C/C | C/C | C/C |  | C = 0.93; T = 0.07 | 0 |
| rps24 |  |  |  |  |  |  |  |  |  |  |  |  |  |  |  |  |  |  |
| 304 | na | G/G | G/G | G/G | G/G | G/G | T/T | G/G | na | G/G | na | G/G | G/G | na | G/G |  | G = 0.91; T = 0.09 | 0 |
| 342 | na | G/G | G/G | G/G | G/G | G/G | C/C | G/G | na | G/G | na | G/G | G/G | na | G/G |  | G = 0.91; C = 0.09 | 0 |
| 391 | na | A/A | A/A | A/A | A/A | A/A | G/G | A/A | na | A/A | na | A/A | A/A | na | A/A |  | A = 0.91; G = 0.09 | 0 |
| 396 | na | T/T | T/T | T/T | T/T | T/T | A/A | T/T | na | T/T | na | T/T | T/T | na | T/T |  | T = 0.91; A = 0.09 | 0 |
| 661 ^b^ | ins/ ins | ins/ ins | −/− | −/− | −/− | ins/ ins | ins/ ins | ins/ ins | na | −/− | −/− | −/− | −/− | na | −/− |  | − = 0.62; ins = 0.38 | 0 |
| ssg (i) | | |  |  |  |  |  |  |  |  |  |  |  |  |  |  |  |  |
| 203 | G/A | A/A | G/G | G/G | G/G | G/G | G/G | G/G | G/G | G/G | G/G | G/G | G/G | G/G | G/G |  | G = 0.90; A = 0.10 | 0.07 |
| EF1a |  |  |  |  |  |  |  |  |  |  |  |  |  |  |  |  |  |  |
| 321 ^c^ | na | na | −/− | −/− | ins/− | −/− | −/− | na | na | na | na | na | na | na | na |  | − = 0.90; ins = 0.10 | 0.2 |
| sTf (ii) |  |  |  |  |  |  |  |  |  |  |  |  |  |  |  |  |  |  |
| 168 | na | na | A/C | A/C | C/C | A/C | A/C | na | na | na | na | na | na | na | na |  | C = 0.6; A = 0.4 | 0.8 |
| 184 | na | na | G/C | G/C | G/C | G/C | G/C | na | na | na | na | na | na | na | na |  | G = 0.5; C = 0.5 | 1.0 |
| 232 | na | na | A/G | A/G | A/A | A/G | A/G | na | na | na | na | na | na | na | na |  | A = 0.6; G = 0.4 | 0.8 |
| 302 | na | na | C/G | C/G | C/C | C/G | C/G | na | na | na | na | na | na | na | na |  | C = 0.6; G = 0.4 | 0.8 |
| 303 | na | na | A/T | A/T | A/A | A/T | A/A | na | na | na | na | na | na | na | na |  | A = 0.7; T = 0.3 | 0.6 |

a – locations of polymorphic sites are referred to by the sites in sequences submitted to GenBank (accession nos [GenBank:DQ834848-DQ834872]).

b – 6-bp indel TCCTCT.

c – 11-bp indel AAAAGAACAAA.
